# Detection of Macrolide and/or Fluoroquinolone Resistance Genes in *Mycoplasma genitalium* Strains Isolated from Men in the Northwest Region of Croatia in 2018–2023

**DOI:** 10.3390/genes15040470

**Published:** 2024-04-08

**Authors:** Sunčanica Ljubin-Sternak, Tomislav Meštrović, Tatjana Marijan, Maja Anušić, Sandra Šuto, Jasmina Vraneš

**Affiliations:** 1 Clinical Microbiology Department, Teaching Institute of Public Health “Dr Andrija Štampar”, 10000 Zagreb, Croatia; tatjana.marijan@stampar.hr (T.M.); maja.anusic@stampar.hr (M.A.); sandra.suto@stampar.hr (S.Š.); jasmina.vranes@stampar.hr (J.V.); 2 Medical Microbiology Department, School of Medicine, University of Zagreb, 10000 Zagreb, Croatia; 3 University Centre Varaždin, University North, 42000 Varaždin, Croatia; tmestrovic@unin.hr; 4 Institute for Health Metrics and Evaluation, University of Washington, Seattle, WA 98195, USA

**Keywords:** *Mycoplasma genitalium*, antimicrobial resistance, molecular testing, resistance genes, macrolides, fluoroquinolones, polymerase chain reaction (PCR), men

## Abstract

*Mycoplasma genitalium* (*M. genitalium*) poses a significant public health challenge due to its association with non-gonococcal urethritis (particularly in men) and antimicrobial resistance. However, despite the prevalence of *M. genitalium* infections and the rise in resistance rates, routine testing and surveillance remain limited. This is the first study from Croatia that aimed to assess the prevalence and trends of resistance in *M. genitalium* strains isolated from male individuals by detecting macrolide and fluoroquinolone resistance genes. The study also aimed to explore the factors associated with resistance and changes in resistance patterns over time. Urine samples collected from male individuals in the Zagreb County and northwest region of Croatia between 2018 and 2023 were tested for *M. genitalium* with the use of molecular methods. Positive samples were subjected to DNA extraction and multiplex tandem polymerase chain reaction (MT-PCR) targeting genetic mutations associated with macrolide (*23S rRNA* gene) and fluoroquinolone (*parC* gene) resistance. Of the 8073 urine samples tested from 6480 male individuals (and following the exclusion of repeated specimens), we found that the prevalence of *M. genitalium* infection was 2.2%. Macrolide resistance was observed in 60.4% of strains, while fluoroquinolone resistance was found in 19.2%. Co-resistance to both antibiotics was present in 18.2% of cases. A statistically significant increase in fluoroquinolone resistance was noted over the study period (*p* = 0.010), but this was not evident for azithromycin resistance (*p* = 0.165). There were no statistically significant differences in resistance patterns between age groups, whereas re-testing of patients revealed dynamic changes in resistance profiles over time. The high burden of macrolide resistance and increasing fluoroquinolone resistance underscore the urgent need for comprehensive resistance testing and surveillance programs. The implementation of resistance-guided treatment strategies, along with enhanced access to molecular diagnostics, is pivotal for effectively managing *M. genitalium* infections.

## 1. Introduction

*Mycoplasma genitalium* (*M. genitalium*) is a sexually transmitted pathogen implicated in 10–35% of non-gonococcal urethritis cases in men [1]. Its unique lack of a cell wall renders traditional antibiotics like β-lactams ineffective, narrowing the treatment options to antibiotic groups such as tetracyclines, macrolides and fluoroquinolones [2]. At the moment, azithromycin is still the recommended first-line treatment against *M. genitalium* [1,3]. However, there has been a notable rise in the occurrence of azithromycin-resistant strains across Europe in recent years [4].

Even though selecting appropriate antibiotics is indispensable for treatment success, the fastidious nature and slow growth of *M. genitalium* render phenotypical antimicrobial susceptibility testing rather impractical [2]. Moreover, resistance to macrolides and fluoroquinolones has been documented since as early as 2010 [5,6]. Despite tetracycline resistance mechanisms not being clearly defined in *M. genitalium*, doxycycline treatment failures exceed 50% of cases [7]. The resistance to other antibiotics is associated with specific chromosomal mutations in *M. genitalium*, leading to treatment failures [8].

Recent advancements in the molecular characterization of the pathogen, exploration of treatment failure cases, and antimicrobial testing in specialized laboratories have led to the identification of mutations associated with resistance in the fastidious-growing bacterial agent. We know that macrolide resistance in *M. genitalium* primarily stems from single base mutations at positions A2058 or A2059 in the *23S rRNA* gene, while fluoroquinolone resistance is linked to mutations in the quinolone resistance-determining region (QRDR) of the *parC* gene, often resulting in amino acid substitutions at positions S83 and D87 [9]. This is why clinical–genetic studies are needed to appraise the exact mechanisms of resistance and inform effective treatment strategies for *M. genitalium* infections.

In 2021, the Centers for Disease Control and Prevention (CDC) recommended a sequential treatment approach in cases where azithromycin resistance testing is unavailable [10]. This involves a seven-day course of doxycycline followed by a week of moxifloxacin. The effectiveness of this resistance-guided approach, incorporating doxycycline and either azithromycin or moxifloxacin based on the presence of macrolide resistance mutations, has been demonstrated [11] and is now part of the European and CDC guidelines. Still, many countries have not implemented this or similar approaches, partly due to a lack of resistance surveillance data and clear protocols.

CE-marked In Vitro Diagnostic Medical Device (CE-IVD) commercial tests for screening antimicrobial resistance in *M. genitalium* are available, but are not part of routine protocols, and they are currently not endorsed by professional societies in Croatia. Furthermore, sexual behaviour data and prior treatment data are often unavailable to laboratories and testing centres, making it challenging to determine the necessity for resistance testing (and is currently not reimbursed as well). All of these factors collectively accentuate the challenges in implementing comprehensive resistance testing protocols and highlight the need for improved access and affordability of such tests.

In this study, we aimed to present the results of the first research endeavour in Croatia that aimed to detect *M. genitalium* macrolide and fluoroquinolone resistance genes in a sample of men from northwest region of Croatia. Additionally, we sought to analyse yearly trends to determine if there has been an increase in resistance to macrolides and fluoroquinolones, evaluate variations in resistance patterns among different age groups of our study sample, understand co-resistance patterns, and examine resistance patterns in repeated isolates. Our study also aimed to correlate the results of the leukocyte esterase test and the number of polymorphonuclear leukocytes (PMNLs) observed per high power field (hpf) under the microscope with the presence of macrolide and fluoroquinolone resistance. With this approach, our goal was to fill a critical gap in our understanding of antimicrobial resistance in *M. genitalium* in Croatia, but also to provide valuable insights that can inform clinical practice and public health policies aimed at combating this sexually transmitted pathogen.

## 2. Materials and Methods

### 2.1. Study Setting and Sampling Approach

**Figure 1:**
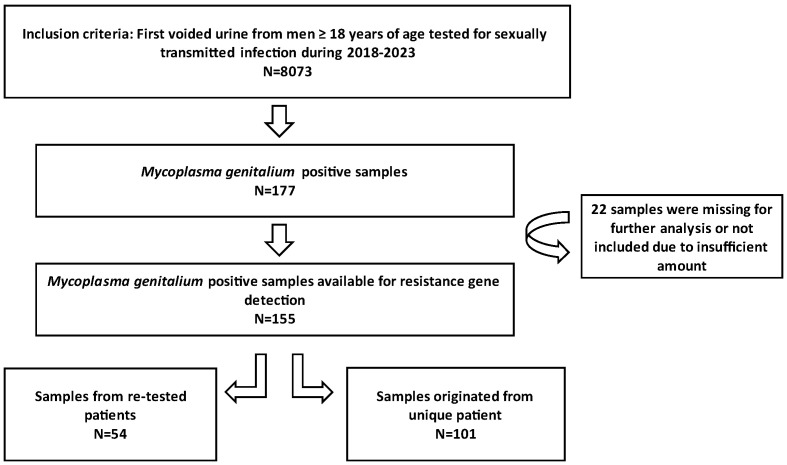
Conceptual framework of the research.

The samples analysed in this study consisted of first voided urine (FVU) collected from male individuals selected by general practitioners in the Zagreb County and northwest region of Croatia. The patients were directed to the Laboratory of the Teaching Institute of Public Health “Dr. Andrija Štampar”, which serves as the Referral Centre for sexually transmitted infection (STI) diagnostics under the Croatian Ministry of Health. All samples submitted to this laboratory for STI diagnostics between 1 January 2018 and 31 December 2023 underwent testing for *M. genitalium*. Positive samples were stored at −80 °C and subsequently tested for macrolide and fluoroquinolone resistance. The conceptual framework of the research is presented in Figure 1. This study received approval from the Ethics Committee of the Teaching Institute of Public Health “Dr. Andrija Štampar” [class: 053-91/23-01/1, number: 251-758-23-10]. Given the anonymity of the patient identities, written informed consent from participants was not required.

### 2.2. Urine Specimen Evaluation and M. genitalium Detection

The rapid dipstick assay for leukocyte esterase (Biognost, Zagreb, Croatia) was employed to identify the presence of esterase enzyme produced by PMNLs in urine samples, which was used to screen for signs of active inflammation. Utilizing a reference colour chart, the dipstick categorized results into negative, 1+ (approximately 70 PMNL/μL), 2+ (approximately 125 PMNL/μL) and 3+ (approximately 500 PMNL/μL). Additionally, the urine sediment was microscopically examined to assess the number of PMNLs per hpf (original magnification: ×400). For analysis purposes, we categorized the results into less than 10 PMNL/μL, 10–25 PMNL/μL and more than 25 PMNL/μL.

*M. genitalium* was identified as part of the routine diagnostics for STI infections using the automated platform ELITe InGenius^®^ (ELITechGroup S.p.a., Torino, Italy). The FTD Urethritis plus kit (Fast Track Diagnostics, Luxembourg) was employed during 2018 and 2019, while the STI PLUS ELITe MGB^®^Kit (ELITechGroup S.p.a., Torino, Italy) was utilized from 2019 until the end of 2023, following the manufacturers’ instructions.

### 2.3. Macrolide and Fluoroquinolone Resistance Gene Detection

*M. genitalium*-positive samples, identified through routine diagnostics, were retrieved from the −80 °C storage, thawed and subjected to DNA extraction using the Nucleic Acid Extraction Kit and the Generotex 96 automated system (XI’an TianLong Science and Technology Co., Ltd., Xi’an, China). The aforementioned system streamlines the extraction process by automating various steps—such as sample preparation, mixing and purification—which ensures consistency and efficiency in DNA extraction across multiple samples.

Following extraction, the samples underwent testing utilizing multiplex tandem polymerase chain reaction (MT-PCR) technology preformed on a High-Plex 24 System (AusDiagnostics, Mascot, NSW, Australia). Briefly, MT-PCR employs two sequential PCR steps: Step 1, a short multiplexed pre-amplification reaction using primers homologous to all targets offered by the kit and Step 2, using primers designed to be ‘nested inside’ the Step 1 primers, which increases the sensitivity and specificity of the assay. For this study, a Urogenital and Resistance 12-well Kit (AusDiagnostics, Mascot, NSW, Australia) was used. This kit is designed to detect eight urogenital pathogens, including *M. genitalium*, and resistance to macrolides/fluoroquinolones. 

In summary, the test was structured to amplify wild-type (i.e., unmutated) sequences of the *23S rRNA* gene for macrolide resistance or the *parC* gene for fluoroquinolone resistance. For strains containing mutations associated with resistance in these regions (A2058 or A2059 for 23S, S83 or D87 for *parC*), a lower amplification efficiency is expected. By comparing the concentrations calculated from the MT-PCR results for each target with a marker elsewhere in the *M. genitalium* genome, the specific identification of mutant strains and consequently the prediction of resistance status was achieved. More specifically, if the concentration of the amplified DNA fragments is lower than that of wild-type sequences, it suggests the presence of resistant strains associated with resistance mutations; conversely, if the concentration of wild-type sequences predominates, it indicates susceptibility to the tested antimicrobial agents.

### 2.4. Data Analysis

Data analysis was performed using R version 4.1.1 (R Foundation for Statistical Computing, Vienna, Austria). Descriptive statistics, inferential analyses and graphical representations were generated to explore and interpret the data. Appropriate statistical tests, such as the chi-square test, were employed to analyse the relationships and differences within the dataset. Statistical significance was set at *p* < 0.05 (two-tailed).

## 3. Results

**Figure 2:**
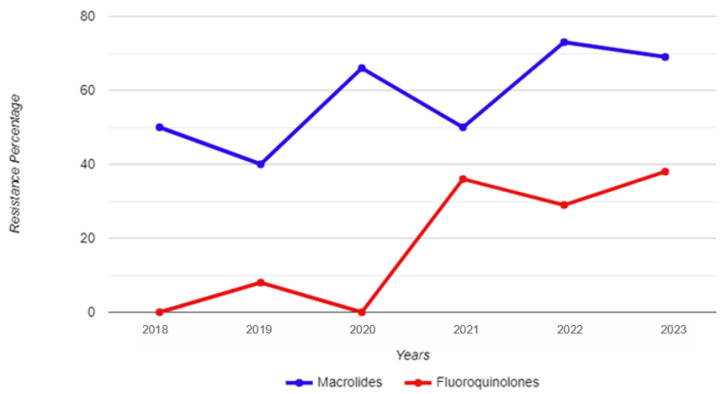
Year-by-year breakdown of macrolide and fluoroquinolone resistance for *Mycoplasma genitalium* strains isolated from urine samples of male study participants.

Between 1 January 2018 and 31 December 2023, a total of 8073 urine samples were tested, originating from 6480 male individuals. Among these samples, 177 tested positive for *M. genitalium*. Thirty-two patients had more than one positive sample, thus giving an overall *M. genitalium* prevalence of 2.2% (145/6480). Subsequently, 155 strains were restored and subjected to testing for resistance to macrolides and fluoroquinolones; of those, 101 were unique patients, and 54 strains were retrieved from 26 re-tested patients (Figure 1). The mean and median age of the *M. genitalium*-positive study participants were 33 and 32 years, respectively, with a range of 19 to 54 (IQR: 9). Table 1 and Figure 2 show the yearly distribution of macrolide and fluoroquinolone resistance in the tested strains. Of note, two data points regarding fluoroquinolone resistance and one data point regarding leukocyte esterase test result/microscopic PMNLs were missing from the dataset.

The total percentage of macrolide resistance in the tested strains was 60.4%, while this percentage was 19.2% for fluoroquinolone resistance. Among all the unique (i.e., not re-tested) strains, the co-occurrence of macrolide and fluoroquinolone resistance was 18.2%. A total of 43.4% of the tested strains were resistant to macrolides only; conversely, there was only one strain resistant to fluoroquinolones that did not show a concurrent resistance to macrolides.

No statistically significant differences were found when comparing the 2018–2020 and 2021–2023 time frames in regard to macrolide resistance (χ^2^ = 1.93; *p* = 0.165); however, a statistically significant increase was observed in fluoroquinolone resistance between these two time periods (χ^2^ = 6.55; *p* = 0.010). A statistical difference was also observed for the prevalence of macrolide and fluoroquinolone co-resistance when comparing these time periods (χ^2^ = 6.12; *p* = 0.013), as only one strain in 2021 harboured only resistance to fluoroquinolones without concomitant resistance to macrolides. There were no statistically significant differences among men 30 years of age and younger, and those older than 30 in regard to either macrolide resistance (χ^2^ = 1.33; *p* = 0.248) or fluoroquinolone resistance (χ^2^ = 0.148; *p* = 0.700) in the isolated *M. genitalium* strains.

A total of 16 and 20 men had a 3+ result on the leukocyte esterase test and more than 25 PMNLs per hpf under the microscope, respectively. However, no statistically significant difference was observed between the macrolide resistance pattern and the results of either the leukocyte esterase screening test (χ^2^ = 0.935; *p* = 0.817) or the number of PMNLs per hpf under the microscope (χ^2^ = 2.85; *p* = 0.241). Similarly, there were no significant differences between the fluoroquinolone resistance pattern and the results of either the leukocyte esterase screening test (χ^2^ = 4.56; *p* = 0.207) or the number of PMNLs per hpf under the microscope (χ^2^ = 2.30; *p* = 0.317).

A total of 26 patients were re-tested. These samples were not included in our prevalence assessment, but they provide important insights into epidemiological trends regarding resistance detection by molecular methods. More specifically, 13 patients were re-tested once, 8 patients twice, 3 of them three times, and finally, 1 patient each underwent re-testing five, six, and eight times, respectively.

**Figure 3:**
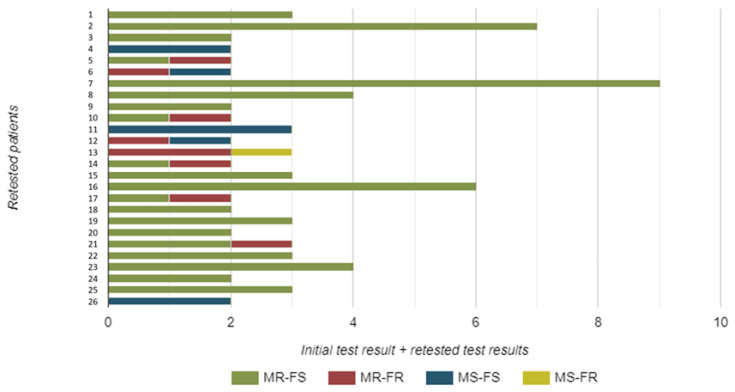
The number and resistance pattern of *Mycoplasma genitalium* strains isolated from urine samples of re-tested male study participants. Legend: MR—macrolide resistant; MS—macrolide sensitive; FR—fluoroquinolone resistant; FS—fluoroquinolone sensitive.

Notably, there were instances where the resistance profile changed (Figure 3). Four strains initially resistant to macrolides showed a change from fluoroquinolone sensitivity to fluoroquinolone resistance in repeated samples, demonstrating dual resistance. One strain showed a change from macrolide sensitivity to macrolide resistance. Another isolate, initially resistant to both macrolides and fluoroquinolones, was now sensitive to macrolides. Similarly, one isolate—initially resistant to both macrolides and fluoroquinolones—exhibited sensitivity to fluoroquinolones upon repeated testing. Finally, one isolate that was initially sensitive subsequently showed dual resistance to both macrolides and fluoroquinolones (Figure 3).

## 4. Discussion

Considering its contribution to the urogenital communicable disease burden in men, monitoring the prevalence of mutations associated with macrolide and fluoroquinolone resistance in *M. genitalium* should be added as an important facet of national public health strategies, particularly in countries and/or regions without any data or where its availability is limited. To our knowledge, this study represents the first exploration of *M. genitalium* resistance genes in male individuals from the Republic of Croatia, shedding light on the overall prevalence of resistant strains in the northwest region of the country. With a total prevalence of macrolide resistance of 60.4% and fluoroquinolone resistance at 19.2%, our results underscore the emergence of antimicrobial resistance as a substantial concern in the management of *M. genitalium* infections in men. The findings also highlight the urgent need for enhanced surveillance and tailored treatment approaches in Croatia to effectively address the issue at hand.

Our results can be compared to recent research endeavours in Europe that covered similar time periods. Among male clients diagnosed with urethritis at the Sexual Health Centre in Amsterdam during the period spanning 2018 to 2019 and with a positive test result for *M. genitalium*, the prevalence rates were high, with 63% exhibiting mutations associated with macrolide resistance, 1% showing mutations linked to fluoroquinolone resistance, and 8% demonstrating multidrug resistance (being resistant to both macrolides and fluoroquinolones) [12]. These figures are substantially higher when compared to the resistance rates observed during the same period in Croatia. Notably, only one strain in Croatia during that specific timeframe exhibited resistance to both macrolides and fluoroquinolones, underscoring the pronounced difference in resistance patterns between the two regions; however, we have demonstrated a significant subsequent rise of both fluoroquinolone resistance and co-resistance in the 2021–2023 time frame.

In a national sentinel surveillance pilot programme conducted in England, a retrospective examination of the data revealed high levels of azithromycin resistance in 2019, with rates reaching 66% among heterosexual men and escalating to 85% among men who have sex with men (MSM) [13]. These figures are higher than the resistance levels observed in our overall sample, as well as our recorded resistance rate for macrolides in 2019, which stood at 40%. A study from Spain interrogated a total of 46 samples that tested positive for *M. genitalium* in the period between 2019 and 2021 [14]. Among these, three mutations were detected in men who have sex with men (MSM) and three mutations were found in men who have sex with women (MSW). The most common mutation observed was the A2059G point mutation, followed by A2058G, A2058T and A2058C [14].

A recent multicentre cross-sectional study conducted to assess the prevalence of mutations associated with macrolide and fluoroquinolone resistance in *M. genitalium*-positive patients in France, spanning the years 2018 to 2020, revealed notable disparities in prevalence rates between male and female individuals [15]. More specifically, across metropolitan France, there was a considerable difference in macrolide resistance prevalence, ranging from 52.4% to 60.2% among men compared to 15.9% to 22.2% among women. Conversely, fluoroquinolone resistance rates ranged from 17.1% to 19.8% in the same geographic region, with no statistically significant differences observed between men and women. Intriguingly, the prevalence of dual resistance-associated mutations was found to be significantly higher among men compared to women during the years 2018 and 2019 [15]. Although this study did not include female patients, the rates in men were comparable to our results, emphasizing the importance of vigilance in monitoring antimicrobial resistance trends and implementing targeted interventions.

Moreover, in a recent study conducted in Belgium, De Baetselier et al. [16] reported a significant prevalence of macrolide-resistant *M. genitalium* across various populations in Belgium. Specifically, among men, 72.4% of the samples analysed in the study exhibited genotypic resistance to macrolides [16]. Despite the study’s limited sample size, all specimens obtained from MSM displayed mutations associated with macrolide resistance, likely attributed to the use of macrolides in treating other STIs or unrelated infections [17]. These treatment protocols, typically involving a single dose of 1 g azithromycin, are suboptimal for *M. genitalium* and may contribute to the development of antimicrobial resistance and the persistence of macrolide-resistant strains [1]. Consequently, in 2022, many countries—including Belgium—adjusted their STI treatment guidelines to minimize the use of azithromycin and thereby mitigate the emergence of azithromycin-resistant *M. genitalium* strains, as well as other STI pathogens [16]. A similar strategic approach may be warranted in Croatia to address the escalating concern of antimicrobial resistance in *M. genitalium* and other STIs.

Seña et al. [18] highlighted how the presence of inflammatory cells might suggest an ongoing inflammatory process stemming from a persistent infection (which may be prompted by resistant microorganisms not amenable to therapy); however, we did not find any significant correlation, which suggests that the elicitation of inflammation and the abundance of inflammatory cells may not be directly associated with the presence of resistance genes. Nonetheless, recognizing the complexity of this relationship, further investigations are necessary to unravel the intricate interplay between resistance mechanisms, potential persistence and the virulence propensity of this microorganism.

From a genetic vantage point, macrolide resistance primarily arises from a single base mutation, typically occurring at position A2058 or A2059 within region V of the *23S rRNA* gene (according to *Escherichia coli* numbering) [19,20]. On the other hand, resistance to fluoroquinolones is associated with mutations found in the quinolone resistance-determining region (QRDR) of the *parC* gene, often resulting in amino acid substitutions at positions S83 and D87 (according to *M. genitalium* numbering) [19,20]. Nonetheless, the existing data supporting correlations between mutations in *parC* and clinical resistance to moxifloxacin are limited. It has been suggested that mutations in the QRDR of the *gyrA* gene in isolates exhibiting a fluoroquinolone-resistant genotype in *parC* may enhance the likelihood of reduced susceptibility to fluoroquinolone antimicrobials [19,20]. Nonetheless, the confirmation of such claims without phenotypic data poses a significant challenge in understanding the intricacies of fluoroquinolone resistance in *M. genitalium* infections.

In any case, this reflects how this problem is approached in the medical literature. For instance, the largest systematic review and meta-analysis conducted on a global scale to date, which examined the prevalence of mutations associated with *M. genitalium* resistance to macrolides and fluoroquinolones, deemed any study design eligible as long as it provided detailed information on the proportions of single-nucleotide polymorphisms (SNPs) at positions 2058 or 2059 of the *23S rRNA* gene, or the amino acid alterations S83R, S83I, D87N, or D87Y in the *parC* gene at baseline or enrolment [9]. More specifically, the selection of changes in *parC* was based on their recognized clinical relevance to moxifloxacin failure. The prevalence of mutations associated with resistance was then determined by summing the samples with at least one key mutation in either the *23S RNA* or *parC* gene (numerator) and dividing by the total number of *M. genitalium*-positive samples successfully characterized for the respective gene (denominator) [9]. Additionally, the prevalence of dual resistance, indicating resistance to both macrolides and fluoroquinolones, was calculated by summing the samples positive for key mutations in both the *23S RNA* and *parC* genes (numerator) and dividing by the total number of *M. genitalium*-positive samples successfully characterized for both genes (denominator) [9].

Looking ahead, targeted sequencing has the propensity to emerge as a robust and potentially high-throughput strategy for identifying genetic mutations associated with drug resistance in *M. genitalium* [20,21], which will also influence further data collection endeavours. A notable advancement in this direction comes from the work of Chiribau et al. [20], who devised a next-generation sequencing (NGS)-based protocol with implications for adaptation in clinical settings, particularly in high-complexity testing laboratories such as public health facilities. Their innovative approach entailed a partial multiplex design involving four PCRs per sample, supplemented by seven primer sets designed to sequence specific regions of the *M. genitalium* genome linked to antimicrobial resistance [20]. This targeted strategy encompasses approximately 0.77% of the entire genome, enabling the comprehensive molecular profiling of genetic determinants associated with resistance to macrolides and fluoroquinolones in a reliable and scalable manner.

For now, research-driven approaches akin to ours will continue to dominate in the literature and be utilized to inform testing and treatment guidelines. Our findings underscore the necessity for reviewing the *M. genitalium* testing guidelines and reimbursement regulations, which focus on conducting testing only in instances of persistent symptoms (as outlined in the European guidelines) [1]. Still, introducing macrolide resistance testing for *M. genitalium*-positive samples could mitigate the utilization of fluoroquinolones and thereby prevent the emergence of multidrug-resistant strains. Almost one-fifth of our samples exhibited resistance-associated mutations against both antimicrobials. While fluoroquinolone resistance may not always lead to treatment failure, certain patients may still respond favourably to moxifloxacin treatment. Nevertheless, instances of clinical multidrug-resistant *M. genitalium* infections are increasingly being documented in many countries [22,23], and here, we observed that this is the emerging problem in Croatia as well. Alternative therapies such as minocycline, pristinamycin and chloramphenicol are considered third-line options [24]; however, accessing these drugs is challenging in Croatia and other European countries.

Therefore, such an imminent loss of effectiveness of macrolides, coupled with the rise and inevitable spread of resistance to fluoroquinolones, underlines the urgent need for novel treatment strategies. While the development of new antimicrobial classes is imperative, there is also ongoing research into antimicrobial combinations aimed at delaying further emergence and dissemination of antimicrobial resistance. A recent study has demonstrated that over 92% of *M. genitalium* infections can be successfully treated, even in populations where two-thirds of cases exhibit resistance to macrolides and 20% of macrolide-resistant cases also show resistance to fluoroquinolones [25]. This successful outcome was achieved through sequential therapy involving pre-treatment with doxycycline followed by the selection of a second antimicrobial based on a macrolide-resistance assay [25]. Substituting azithromycin with doxycycline as the initial treatment for *M. genitalium* offers a dual benefit: it reduces the overall azithromycin usage while also decreasing the *M. genitalium* load. However, its pervasive usage is still a matter of discussion.

With the increasing availability of multiplex testing for screening STIs in Croatia, there is an urgent requirement for updated recommendations regarding *M. genitalium* testing and treatment. Our data could significantly influence these guidelines. Despite the small sample size, our study suggests that conducting resistance testing may be prudent to avoid potential treatment failures (as evidenced in our study with a large number of repeated samples). The use of combined assays for the simultaneous detection of *M. genitalium* and resistance mutations to enhance antimicrobial stewardship and curb the spread of resistance could be a way forward. Additionally, our study underscores the importance of conducting antimicrobial resistance surveillance in *M. genitalium* at the local level (considering, at the same time, the available laboratory infrastructure and economic ramifications).

In any case, continuous sentinel surveillance should be seen as vital for informing updates to national management guidelines, while the future integration of combined molecular-based assays detecting *M. genitalium* and resistance genes will greatly facilitate individualized therapy. This diagnostic approach, along with sequential therapy, can be observed as essential to impede the inevitable progression to multidrug-resistant, untreatable *M. genitalium*. Further research is warranted to comprehend and quantify the relationship between the presence of resistance mutations and treatment failure.

This study was subject to certain limitations. We primarily relied on urine samples collected from male individuals in one specific region of the Republic of Croatia, limiting the generalizability of the findings to broader populations or other demographic groups. There is also a possibility of a selection bias, as tested individuals may have sought medical attention for specific symptoms. Notably, there is a lack of clinical and epidemiological data, as well as information regarding patients’ sexual practices, which could provide valuable insights for understanding the context and factors influencing antimicrobial resistance. The patients who underwent re-testing might have been infected with either the same or a different strain of *M. genitalium* on each occasion, which we could not differentiate. Methodological constraints confined our assessment of fluoroquinolone mutations solely to the *parC* gene, overlooking potential mutations in the *gyrA* gene. While mutations in the *gyrA* gene are less commonly reported and typically occur in conjunction with mutations in the *parC* gene, it is essential to recognize that the occurrence of non-synonymous mutations in both genes in *M. genitalium* can exacerbate fluoroquinolone resistance, as discussed previously. Hence, there is a critical need to monitor mutations in both the *gyrA* and *parC* genes. The treatment details were unavailable for any of the patients, particularly those with repeated samples, and there was a lack of follow-up information regarding the potential effectiveness of treatment. Most of these limitations stem from the retrospective nature of the study.

## 5. Conclusions

Our data indicate a high prevalence of mutations associated with macrolide and fluoroquinolone resistance that has already surpassed the WHO’s 5% threshold for changing empirical antimicrobial therapy for gonorrhoea, with 18.2% of strains exhibiting co-resistance to both antimicrobial groups. While fluoroquinolone resistance significantly increased over time, no significant change was noted for azithromycin resistance, and the re-testing of patients revealed dynamic changes in resistance profiles over time. This reveals the necessity to move away from the widespread use of single-dose azithromycin in treating STI syndromes, while the adoption of new antimicrobial classes (and possibly combination therapy) will be essential to enhance treatment efficacy. Meanwhile, we believe substantial progress can be made in reducing the spread of resistant *M. genitalium* by pursuing similar research endeavours, curtailing the use of macrolides, employing resistance-guided treatment strategies, and establishing national and international surveillance programmes for *M. genitalium* and antimicrobial resistance.

## Figures and Tables

**Table 1:** A detailed breakdown showing the levels of *Mycoplasma genitalium* resistance to macrolides and fluoroquinolones among men tested between 2018 and 2023.

	Resistance to Macrolides			Resistance to Fluoroquinolones		
**Year**	Yes	No	Total	Yes	No	Total
**2018**	3	3	6	0	6	6
**2019**	6	9	15	1	13	14
**2020**	6	3	9	0	9	9
**2021**	10	10	20	5	14	19
**2022**	16	6	22	5	17	22
**2023**	20	9	29	8	21	29
**Total**	**61**	**40**	**101**	**19**	**80**	**99**

## Data Availability

The original dataset is available on reasonable request from the corresponding author.
